# Jianpi Jiedu Xiaozheng Fang Regulates Hepatocellular Carcinoma Proliferation and Metastasis Based on Network Pharmacology

**DOI:** 10.1111/jcmm.71040

**Published:** 2026-03-07

**Authors:** Bin Li, Han‐Qian Shi, Rui Luo, Zi‐Qi Zhang, Xiao‐Chen Dong, Xiao‐Hua Li, Shi‐Qin Ye, Chong Zhong

**Affiliations:** ^1^ Guangzhou University of Chinese Medicine Guangzhou China; ^2^ First Clinical Medical College Guangzhou University of Chinese Medicine Guangzhou China; ^3^ Beijing University of Chinese Medicine Shenzhen Hospital (Longgang) Shenzhen China; ^4^ Department of Biliary‐Pancreatic Surgery The First Affiliated Hospital of Guangzhou University of Chinese Medicine Guangzhou China

**Keywords:** BIRC5, hepatocellular carcinoma, hippo pathway, JPJDXZF, organoid

## Abstract

Hepatocellular carcinoma (HCC) is a primary malignant tumour that impacts patients' quality of life. Currently, clinical experience from The First Affiliated Hospital of Guangzhou University of Chinese Medicine suggests that Jianpi Jiedu Xiaozheng Fang (JPJDXZF) demonstrates promising efficacy in the treatment of HCC. We aimed to explore the mechanisms of JPJDXZF in HCC based on network pharmacology. The components and their relevant targets of JPJDXZF were identified using databases such as SymMap, TCMID, TCMSP, and TCM‐ID. Following ADME screening, 1443 active components of JPJDXZF were identified, and 435 corresponding drug targets were predicted using the SwissTargetPrediction database. Subsequently, prognosis‐related differentially expressed genes (DEGs) associated with HCC were analyzed using TCGA and GTEx datasets, and a gene expression matrix was derived. Key genes involved in HCC regulation were identified, and functional analyses were performed. Furthermore, we explored the regulatory effects of JPJDXZF at the cellular, organoid, and animal levels. We identified 18 intersecting genes between HCC prognosis‐related genes and JPJDXZF‐target genes. Venn diagram analysis successfully identified BIRC5 and CYP2E1 as two potential targets for JPJDXZF in treating HCC. Pathway enrichment analysis indicated that the core targets of JPJDXZF were enriched in multiple signalling pathways, including the Hippo pathway, in which BIRC5 is involved as a downstream regulatory gene. In in vitro experiments, JPJDXZF‐containing serum significantly reduced the viability and migration of HepG2 and MHCC97‐H cells, leading to a decrease in organoid diameter and ATP activity in HCC organoids. In in vivo experiments, tumours in nude mice treated with JPJDXZF exhibited reduced volume and weight, along with decreased expression of BIRC5 and Hippo pathway effectors YAP and TAZ. At the mechanistic level, JPJDXZF treatment was associated with altered Hippo pathway–related signalling, accompanied by reduced YAP/TAZ activity and changes in BIRC5 expression, together with effects on HCC cell proliferation and apoptosis. In addition, siMST1/2 interference and EMT inhibitor‐1 treatment partially attenuated the effects of JPJDXZF on cell viability, migration, and apoptosis. JPJDXZF regulates BIRC5 expression in association with Hippo pathway activity in HCC. In vitro, in vivo, and molecular mechanism analyses support JPJDXZF as a potential therapeutic strategy for HCC by modulating key proteins in the Hippo pathway, thus affecting HCC cell proliferation, apoptosis, and migration.

**Abbreviations**
ANOVA: analysis of variance
BHSSC: Baihuasheshecao
BJ: Biejia
BZ: Baizhu
CCK‐8: Cell Counting Kit 8
DEGs: differentially expressed genes
DMEM: Dulbecco's Modified Eagle's Medium
DO: Disease Ontology
DS: Dangshen
EZ: Ezhu
FL: Fuling
GC: Gancao
GEO: Gene Expression Omnibus
H&E: haematoxylin–eosin
HCC: hepatocellular carcinoma
IHC: immunohistochemistry
JPJDXZF: Jianpi Jiedu Xiaozheng Fang
KEGG: Kyoto Encyclopedia of Genes and Genomes
ME: Module Eigengene
NP: network pharmacology
QH: Qinghao
qRT‐PCR: quantitative real‐time PCR
ROC: received operating characteristic
TBC: Tubiechong
TCM: Traditional Chinese medicine
TOM: topological overlap matrix
TR: Taoren
WGCNA: weighted gene co‐expression network analysis
XKC: Xiakucao

## 1 Introduction

Hepatocellular carcinoma (HCC) is an increasingly prevalent malignancy with a continuously rising mortality rate, posing a major global health burden [1, 2]. Owing to its high recurrence rate and limited therapeutic options, the 5‐year survival rate of HCC remains approximately 10% [3]. The development of HCC is driven by multiple genetic, environmental, and behavioural factors, including cirrhosis, alcoholic liver disease, diabetes, and obesity [4, 5]. Although surgical resection and liver transplantation remain the primary curative treatments, therapeutic decision‐making is largely based on tumour burden and staging systems, which fail to fully account for the pronounced biological heterogeneity of HCC [6, 7]. Therefore, elucidating the molecular mechanisms underlying HCC progression and identifying novel therapeutic strategies remain urgent clinical needs.

Traditional Chinese medicine (TCM) represents a rich repository of therapeutic agents derived from long‐term clinical practice [8]. Due to its multi‐component, multi‐target, and multi‐pathway characteristics, TCM has attracted increasing attention in cancer research, particularly for its low toxicity and holistic regulatory effects [9, 10]. Accumulating evidence suggests that TCM‐based therapies can improve liver function, inhibit tumour progression, and reduce postoperative recurrence in HCC, thereby offering distinct advantages as complementary treatments [11, 12]. Herbal formulas based on the principles of ‘Yiqi Jianpi’ (replenishing qi and strengthening the spleen) and ‘Jiedu Huayu’ (detoxification and removal of blood stasis) have shown therapeutic efficacy in HCC management [13, 14]. Previous studies have reported that the potential targets of Jianpi Jiedu‐related prescriptions are mainly involved in metabolic regulation and apoptosis in HCC [15]. However, the molecular mechanisms underlying the antitumor effects of Jianpi Jiedu Xiaozheng Fang (JPJDXZF) have not been systematically elucidated.

Notably, based on clinical practice at The First Affiliated Hospital of Guangzhou University of Chinese Medicine, JPJDXZF has demonstrated promising therapeutic effects in patients with advanced or massive HCC, providing a strong clinical rationale for mechanistic investigation.

Given the complexity of TCM formulations, network pharmacology (NP) has emerged as an effective strategy for systematically deciphering the interactions among active compounds, molecular targets, and disease pathways [16, 17, 18]. By integrating pharmacological data with bioinformatics analysis, NP enables the construction of ‘compound‐target‐pathway‐disease’ networks and is particularly suitable for investigating the multi‐target mechanisms of TCM formulas in complex diseases such as HCC [19]. Therefore, NP provides a rational framework for exploring the potential molecular basis of JPJDXZF in HCC treatment.

Mechanistically, dysregulation of the Hippo signalling pathway has been increasingly implicated in HCC progression [20, 21]. The transcriptional coactivators YAP and TAZ, as core downstream effectors of this pathway, promote tumour growth by activating TEAD‐dependent transcriptional programs that regulate cell proliferation and apoptosis resistance [22]. Importantly, emerging experimental evidence has demonstrated that activation of YAP/TAZ signalling can upregulate the anti‐apoptotic gene BIRC5 (Survivin), thereby enhancing cell survival, whereas inhibition of YAP/TAZ activity leads to reduced BIRC5 expression [23]. These findings provide direct experimental support for a functional YAP/TAZ‐BIRC5 regulatory axis in cancer.

In this study, we combined NP and bioinformatics analyses with cellular, patient‐derived organoid, and in vivo experiments to systematically investigate the antitumor effects and molecular mechanisms of JPJDXZF in HCC, with a particular focus on the Hippo‐YAP/TAZ‐BIRC5 signalling axis. Our findings aim to provide a mechanistic basis for the therapeutic application of JPJDXZF and offer insights for the development of novel HCC treatment strategies.

## 2 Materials and Methods

### 2.1 JPJDXZF Targets Identification and Network Establishment

We used SymMap, TCMID, TCMSP (https://tcmspw.com/tcmsp.php), and TCM‐ID databases to search for ADME parameter information of the active components of JPJDXZF and included those with a DL value of ≥ 0.18 for target prediction. The included compounds were then subjected to target prediction using SwissTargetPrediction database (http://www.swisstargetprediction.ch/), and targets with a possibility greater than 0 were ultimately included. The Cytoscape 3.7.2 software was employed to construct the JPJDXZF‐active component‐target network.

### 2.2 Screening of Differentially Expressed Genes (DEGs) Associated With HCC

Clinical and transcriptome data associated with HCC were retrieved from the TCGA and GTEx databases. R software was employed to manage, synthesise, and analyse the clinical data, leading to the derivation of a gene expression matrix related to JPJDXZF targeting HCC. DEGs were identified in the dataset using a threshold of *p* < 0.05. Using the ggpubr package, the expression of these DEGs in HCC samples was analysed, and a boxplot of HCC‐JPJDXZF related gene expression was created.

### 2.3 Functional Analysis and the Intersection of Survival Prognostic Genes of TCGA/GTEx and JPJDXZF Target Genes

Bioconductor package, clusterProfiler package, and DOSE package in R language were utilised to perform Disease Ontology (DO) enrichment analysis on the potential targets of JPJDXZF. The online analysis tool jvenn (http://jvenn.toulouse.inra.fr/app/example.html) was used to obtain the intersection between survival prognosis genes from TCGA/GTEx and HCC‐JPJDXZF target genes.

### 2.4 Acquisition of HCC Disease Targets

Transcriptome chip data related to HCC were retrieved from the Gene Expression Omnibus (GEO) database. The search criteria were limited to data containing the keyword ‘Hepatocellular Carcinoma’ and were specific to the human species. Our study utilised the microarray dataset GSE138178 (which included tumour tissue samples from 49 HCC patients and 49 paired adjacent tissue samples) as an external test set for validation. The data were preprocessed using the Bioconductor package in R, which involved background correction, normalisation, and calculation of expression values. Next, the limma package was utilised to identify DEGs between the two sample groups, with criteria set at *p* < 0.05 and a fold change of at least 2 (|log_2_FC| ≥ 1). Based on these criteria, upregulated and downregulated DEGs were identified. The heatmap package was then employed to visualise and conduct cluster analysis on the selected DEGs.

### 2.5 Construction of Weighted Gene Co‐Expression Network

In our gene expression analysis of HCC, we initially excluded genes with expression levels above the fourth quartile of all variances. The remaining genes were then input into the ‘Weighted gene co‐expression network analysis (WGCNA)’ package in R to build a weighted gene co‐expression network for HCC. Through sample clustering analysis and a scale‐free network model, the optimal soft threshold β for the network was determined, and based on this, a gene adjacency matrix was constructed. Additionally, the topological overlap matrix (TOM) was employed to measure the similarity between genes, and a hierarchical clustering tree was constructed using this matrix. Gene modules were identified and merged using the dynamic hybrid cutting method, resulting in a gene dendrogram. Following module division, the Module Eigengene (ME) for each module was calculated and correlated with the clinical features of HCC patients. Pearson correlation coefficient was utilised to evaluate the association between ME and clinical traits, and the module most closely related to HCC was determined as the key hub module. Genes within this module were then further filtered for detailed analysis.

### 2.6 Core Target Relative Expression, Received Operating Characteristic (ROC) Evaluation and Enrichment Analysis

In the in‐depth analysis of the molecular mechanisms of HCC, we compared the DEGs related to the prognosis of HCC treated with JPJDXZF obtained earlier with the HCC‐GSE138178 related genes and the HCC‐GSE138178‐WGCNA related genes. These were imported into the online Venn diagram tool InteractiVenn to identify potential targets for JPJDXZF in treating HCC. To further investigate whether core genes affect the survival of HCC patients, the Kaplan–Meier Plotter tool was utilised for survival prognosis analysis of core genes. R packages survival, caret, glmnet, survminer, and survivalROC were utilised to plot ROC curves for HCC core genes, thereby assessing their potential and accuracy in disease diagnosis. Additionally, the Bioconductor and clusterProfiler packages in R were used to perform Kyoto Encyclopedia of Genes and Genomes (KEGG) pathway enrichment analysis on core targets of JPJDXZF in treating HCC, and the pathview package was employed to draw the corresponding signal pathway diagrams.

### 2.7 Collection of Clinical Samples

We collected HCC tissue and adjacent tissue samples from patients diagnosed with HCC through imaging, serological, or histopathological examination at The First Affiliated Hospital of Guangzhou University of Chinese Medicine. Patients with non‐HCC confirmed pathologically and those lacking complete clinical data were excluded. Fresh HCC tumour tissue samples were collected, placed in tissue preservation solution (Guangzhou Woggen Biotechnology Co. LTD), and stored at 4°C for further research. Written informed consent was obtained from all patients who provided samples, and the study was approved by The First Affiliated Hospital of Guangzhou University of Chinese Medicine (approval number KY‐2024‐369, approval date 20241105). All procedures performed in studies involving human participants were in accordance with the ethical standards of the institutional and/or national research committee and with the 1964 Helsinki Declaration and its later amendments or comparable ethical standards.

### 2.8 Composition of JPJDXZF

JPJDXZF is a TCM formula composed of the following herbal materials per dose: *Codonopsis pilosula* (Franch.) Nannf. (Dangshen, 10 g), Poria cocos (Schw.) Wolf (Fuling, 5 g), Atractylodes macrocephala Koidz. (Baizhu, 5 g), Dioscorea opposita Thunb. (Shanyao, 10 g), Curcuma phaeocaulis Valeton (Ezhu, 5 g), Eupolyphaga sinensis Walker (Tiebiechong, 5 g), *Prunus persica* (L.) Batsch (Taoren, 5 g), *Trionyx sinensis* Wiegmann (Biejia, 5 g), *Artemisia annua* L. (Qinghao, 5 g), Cremastra appendiculata (D. Don) Makino (Shancigu, 5 g), Scutellaria barbata D. Don (Banzhilian, 10 g), *Prunella vulgaris* L. (Xiakucao, 5 g), Glycyrrhiza uralensis Fisch. (Gancao, 5 g), and Hedyotis diffusa Willd. (Baihuasheshecao, 5 g). All herbal materials were mixed according to the prescribed ratio and used for subsequent preparation of JPJDXZF‐containing serum. All experiments were performed using herbal materials from the same production batch.

### 2.9 Preparation of JPJDXZF‐Containing Serum

Six‐week‐old male Sprague–Dawley (SD) rats were used for the preparation of JPJDXZF‐containing serum. The animals were randomly divided into a control group (*n* = 3) and a JPJDXZF‐treated group (*n* = 3). Rats in the treatment group were administered JPJDXZF by oral gavage at a dose of 1.53 g per rat per day, while control animals received an equal volume of distilled water. All treatments were performed once daily for seven consecutive days. At the end of the treatment period, rats were anaesthetised with 4% isoflurane, and blood samples were collected from the abdominal aorta. The collected blood was centrifuged at 1000 g for 10 min to obtain serum. Serum samples from animals within the same group were pooled. The same pooled serum batch was used for all in vitro experiments to avoid batch‐to‐batch variation. Serum aliquots were stored at −20°C until use.

### 2.10 Cell Culture and Identification of JPJDXZF‐Containing Serum Toxicity

Human HCC cells HepG2 (CTCC‐001‐0014, Meisen) and MHCC97‐H (CTCC‐400‐0192, Meisen) were cultured in Dulbecco's Modified Eagle's Medium (DMEM, C11965500BT, GIBCO) with 10% FBS (16000‐044, GIBCO) and 1% Penicillin/Streptomycin solution (BL505A, Biosharp) at 37°C, 5% CO_2_, and saturated humidity in an incubator. HepG2 and MHCC97‐H cells were cultured and treated with JPJDXZF‐containing serum at concentrations of 0%, 1%, 5%, 10%, and 30%. After 24 h, the IC50 was evaluated using Cell Counting Kit 8 (CCK‐8) assay to select optimal treatment concentration.

### 2.11 Cell Treatment

HepG2 and MHCC97‐H cells were treated with JPJDXZF‐containing serum, with groups designated as control (culturing HepG2 or MHCC97‐H cells and treating with control serum) and JPJDXZF (culturing HepG2 or MHCC97‐H cells and treating with JPJDXZF‐containing serum). BIRC5 was overexpressed by transfecting plasmids that overexpress BIRC5 into HepG2 and MHCC97‐H cells, with groups divided as: NC and BIRC5‐OE. Additionally, EMT inhibitor‐1 (Selleckchem, S7279) was used as a pharmacological probe to modulate Hippo pathway‐related signalling. This compound was originally identified as C19 and has been reported to modulate the MST/LATS‐YAP/TAZ axis, partly through activation of MST/LATS kinases and AMPK signalling, thereby promoting TAZ degradation. Given that EMT inhibitor‐1 has been shown to affect multiple signalling pathways [24], it is not considered a Hippo‐specific inhibitor and was therefore used as a supportive pharmacological tool in this study. EMT inhibitor‐1 was added to treat HepG2 and MHCC97‐H cells, which were further divided into the following groups: control (culturing HepG2 or MHCC97‐H cells and treating with control serum), JPJDXZF (culturing HepG2 or MHCC97‐H cells, transfecting with NC plasmid, and then treating with JPJDXZF‐containing serum), JPJDXZF + EMT inhibitor‐1 (culturing HepG2 or MHCC97‐H cells, transfecting with NC plasmid, and then treating with JPJDXZF‐containing serum and EMT inhibitor‐1), and JPJDXZF + BIRC5‐OE (culturing HepG2 or MHCC97‐H cells, transfecting with BIRC5‐OE plasmid, and then treating with JPJDXZF‐containing serum).

For siMST1/2 interference, small interfering RNAs targeting MST1/2 (siMST1/2) and negative control siRNA (si‐NC) were synthesised by GenePharma (Shanghai, China). HepG2 and MHCC97‐H cells were transfected with siMST1/2 or si‐NC using Lipofectamine 2000 (11668‐027, Invitrogen) for 48 h. Following cell digestion, the cells (2 × 10^4^ cells/well) were plated into 24‐well plates and incubated overnight at 37°C with 5% CO_2_. On the following day, when the cells reached 80% confluence, transfection was carried out for 48 h following the protocol for Lipofectamine 2000 (11668‐027, Invitrogen).

### 2.12 Organoid Culture and Treatment and ATP Detection

The isolation and culture of HCC organoid was based on previous reference [25]. The HCC organoids were established from tumour tissue obtained from a 66‐year‐old male patient diagnosed with grade III HCC. The organoid growth was observed daily, and the culture medium was changed every 2–3 days. Organoids derived from the same patient were used for subsequent experiments, and organoid‐based assays were performed with three independent biological replicates. The HCC organoid culture medium was replaced with medium containing control serum or JPJDXZF‐containing serum and continued to be cultured for 9 days, observing the organoid growth. Additionally, the ATP activity of the organoids was measured. An equal volume of CellCounting‐Lite 3D (DD1102, Vazyme) that had been balanced to room temperature was added to the cell culture to be tested. The mixture was vortexed vigorously for 5 min to fully lyse the cell aggregates. It was then allowed to stand at room temperature for 25 min to stabilise luminescent signal before detection.

### 2.13 In Vivo Tumorigenesis

Twelve 4‐week‐old SPF‐grade BALB/c nude mice, weighing between 14 and 16 g, were randomly assigned to a control group and a JPJDXZF group, with six mice in each group. After the mice are adapted to the environment, a suspension of transfected HepG2 cells (3 × 10^6^) was inoculated subcutaneously to establish an HCC animal model. For the control group mice, after the subcutaneous injection of HepG2 cells formed a tumour with an average volume of 85 mm^3^, they were administered an equal volume of distilled water daily for 15 days. For the JPJDXZF group mice, after the subcutaneous injection of HepG2 cells formed a tumour with an average volume of 85 mm^3^, they were gavaged with JPJDXZF once a day for 15 days. At the conclusion of the experiment, after euthanasia with CO_2_, the tumour tissues were removed for further experiments. All animal experiments have been approved by Guangzhou Forevergen Medical Laboratory Animal Center (approval number IACUC‐AEWC‐F240601016, approval date 20240601). Animal experiments were performed in accordance with ARRIVE guidelines and the IACUC Handbook (third edition).

### 2.14 CCK‐8 Assay

HepG2 and MHCC97‐H cells were rinsed once with PBS, and 0.25% Trypsin–EDTA (1×) (25200‐072, GIBCO) was digested into a single‐cell suspension. After digestion was terminated by complete medium, the cells were re‐suspended by complete medium. Cells were counted, and the concentration was adjusted to 3 × 10^4^ cells/mL. They were then seeded into 96‐well plates at 100 μL per well, which equated to 3 × 10^3^ cells per well. For each cell line, three replicate wells were tested daily, with 100 μL of complete medium added to each well. Cells were cultured at 37°C and 5% CO_2_. After 24 or 48 h, CCK‐8 reagent (G4103, Servicebio) was added at a ratio of 1:10, meaning 10 μL of the test solution was added to 100 μL of the culture solution. After incubating at 37°C for 2 h, absorbance at 450 nm was measured using a microplate reader.

### 2.15 Cell Apoptosis

Apoptosis in HepG2 and MHCC97‐H cells was assessed using Annexin V‐APC/PI Apoptosis Kit (E‐CK‐A217, Elabscience). Following treatment as per the experimental design, cells were centrifuged at 300 *g* for 5 min, supernatant was removed, and cells were collected, washed once with PBS, and gently resuspended for counting. A suspension of 1–5 × 10^5^ cells was taken, centrifuged at 300 *g* for 5 min, and supernatant was discarded. Cells were then washed with PBS, centrifuged again to remove supernatant, and resuspended in 500 μL of diluted 1 × Annexin V Binding Buffer. Suspension was supplemented with 5 μL of Annexin V‐APC Reagent and 5 μL of PI Reagent (50 μg/mL). After gentle mixing, cells were incubated at room temperature for 15–20 min in the dark, and flow cytometry was performed immediately.

### 2.16 Transwell Migration Assay

HepG2 and MHCC97‐H cells were rinsed with PBS and then dissociated into a single‐cell suspension using 0.25% trypsin. A portion of the cell suspension was centrifuged at 800 rpm for 5 min, and supernatant was removed. Cells were then resuspended in basal medium, counted, and adjusted to a concentration of 1 × 10^6^ cells/mL. 100 μL of cell suspension were added to the upper chamber of a Transwell insert, while 600 μL of complete medium was added to the lower chamber. After a 24‐h incubation in a cell culture incubator, cells were washed three times with PBS. A cotton swab was used to remove cells from the upper surface of the upper chamber. 2 mL of fixative were added to the well plate, and the Transwell was placed inside to fix for 15 min. Cells were then washed three times with PBS. 1 mL staining solution (BL710A, Biosharp) was added to the well plate, ensuring that the Transwell was immersed in the liquid, and the cells were stained for 10 min. The staining solution was discarded, and 1 mL colour adjustment solution A was added, followed by one drop of colour adjustment solution B. The mixture was quickly mixed and observed under a microscope.

### 2.17 Scratch Assay

HepG2 and MHCC97‐H cells were treated with trypsin and subsequently plated into a 6‐well plate at a density of 1 × 10^6^ cells per well. Once the cells had fully adhered, medium was replaced and mitomycin C was introduced at a final concentration of 1 μg/mL for 1 h to prevent cell division. A scratch was made along the edge of the lid using a 200 μL pipette tip, perpendicular to the plate bottom. Cells were rinsed three times with PBS to eliminate scratched cells, and basal medium was added. Cells were then returned to the incubator for further culturing. At 0 and 48 h, eight random scratch areas were imaged. The distances were measured using Image‐J software, and the migration rate was calculated as (0 h—other time points)/0 h.

### 2.18 Quantitative Real‐Time PCR (qRT‐PCR)

Initially, total RNA was isolated using TriQuick Reagent Total RNA Extraction Kit (R1100, Solarbio). Then, the RNA was converted into cDNA using the 5 × RT SuperMix for qPCR (K1074, APEXbio). The gene expression levels were detected using 2 × SYBR Green qPCR Master Mix (K1070, APEXbio) on the iQ5 Multicolor Real‐Time PCR Detection System (582BR 005500, BIO‐RAD). β‐actin served as the internal reference gene, and relative expression was determined using the 2^−ΔΔCt^ method. The primer sequences utilised in this study are as follows: H‐BIRC5‐F: CAGCCCTTTCTCAAGGACCA, H‐BIRC5‐R: TGTTCCTCTATGGGGTCGTC; H‐YAP‐F: TCCCGGGATGTCTCAGGAAT, H‐YAP‐R: CCCAGGAATGGCTTCAAGGT; H‐TAZ‐F: TGGAACCTGAAGTTGATGCG, H‐TAZ‐R: ACACCTTTCCCCTCATTCTCTG; H‐GAPDH‐F: CATCATCCCTGCCTCTACTGG, H‐GAPDH‐R: GTGGGTGTCGCTGTTGAAGTC.

### 2.19 Nuclear and Cytoplasmic Protein Extraction

Nuclear and cytoplasmic proteins were isolated using a commercial nuclear and cytoplasmic extraction kit, following the manufacturer's instructions. After fractionation, nuclear and cytoplasmic extracts were collected separately and subsequently used for Western blot analysis to evaluate the subcellular localisation of YAP and TAZ.

### 2.20 Western Blot

Total protein was extracted from cells or tumour tissues using RIPA (P0013B, Beyotime). After quantification with a BCA kit (BL521A, Biosharp), the target proteins were detected using a 12% polyacrylamide gel with a loading amount of approximately 20 μg. Total protein was separated by SDS‐PAGE and transferred to a PVDF membrane. The membrane was then blocked with 5% skim milk and incubated overnight at 4°C with primary antibodies: BIRC5 (Proteintech, 10778‐1‐AP, 1:1000), YAP (Proteintech, 13663‐1‐AP, 1:1000), TAZ (Proteintech, 10670‐1‐AP, 1:1000), GAPDH (Proteintech, 60004‐1‐Ig, 1:5000), Phospho‐LATS1 (Thr1079) (Proteintech, 28998‐1‐AP, 1:1000), Phospho‐YAP1 (Ser127) (Proteintech, 80694‐2‐RR, 1:1000), Phospho‐MST1 (Thr183)/MST2 (Thr180) (Proteintech, 80093‐1‐RR, 1:1000), Phospho‐WWTR1 (Ser89) (Invitrogen, PA5‐105066, 1:1000), MST1 (Proteintech, 22245‐1‐AP, 1:1000), and STK3/MST2 (Proteintech, 12097‐1‐AP, 1:1000). It was subsequently incubated with HRP‐conjugated secondary antibodies. The membrane was then exposed to ECL chemiluminescent solution (K‐12045‐D50, Advansta), and target proteins were visualised using ChemiScope6100 (CLiNX). Band intensities were quantified using ImageJ software (version 1.53, National Institutes of Health, USA).

### 2.21 TEAD Luciferase Reporter Assay

TEAD transcriptional activity was measured using a dual‐luciferase reporter assay system (Promega, E1910). HepG2 and MHCC97‐H cells were co‐transfected with the TEAD luciferase reporter plasmid and Renilla luciferase plasmid (Promega, E1910) using Lipofectamine 2000 (Invitrogen, 11668‐027) according to the manufacturer's instructions. After treatment, luciferase activity was measured using a luminometer, and the Firefly luciferase signal was normalised to the Renilla luciferase signal to account for differences in transfection efficiency.

### 2.22 Haematoxylin–Eosin (H&E) Staining

The morphology of organoids and tumour tissues was examined using an H&E staining kit (G1100, Solarbio). The organoids and tumour tissues were decalcified, fixed with formaldehyde, dehydrated, and embedded in paraffin. They were then cut into 5 μm sections. Sections were dewaxed to water using an ethanol gradient (75%–100%), stained with haematoxylin for 1 min, rinsed with distilled water, and blued with PBS. They were subsequently stained with eosin for 0.5 min and rinsed with distilled water. Sections were then dehydrated with an ethanol gradient (95%–100%), with each step lasting 5 min. After removal, sections were placed in xylene for 10 min, twice, and then mounted with neutral resin for microscopic observation using a microscope (BA410T, Motic).

### 2.23 Immunohistochemistry (IHC)

IHC detection was employed to assess Arg‐1, CK18, and Ki67 levels in organoids and tumour tissues. Sections were baked at 60°C for 12 h, dewaxed to water, and antigens were retrieved by heat. A 1% periodic acid solution was applied for 10 min at room temperature to inactivate endogenous enzymes. Primary antibodies for Arg‐1, CK18, and Ki67 were incubated overnight at 4°C. Secondary antibodies were incubated at 37°C for 30 min. DAB was used for colour development, and sections were counterstained with haematoxylin for 5–10 min, and blued with PBS. Sections were dehydrated with an alcohol gradient (60%–100%), with each step lasting 5 min. After removal, sections were placed in xylene for 10 min, twice, and then mounted with neutral resin for microscopic observation.

### 2.24 Statistical Analysis

Statistical analyses were performed using GraphPad Prism (version 9.5). Data are presented as mean ± SD. The experiments were performed with three independent biological replicates. Comparisons between two groups were conducted using an unpaired two‐tailed Student's *t*‐test. For comparisons among multiple groups, one‐way ANOVA was applied followed by appropriate post hoc multiple‐comparison tests. IC_50_ values were calculated by nonlinear regression using a four‐parameter logistic model. *p* < 0.05 was considered statistically significant.

## 3 Results

### 3.1 Prognosis‐Related and Key Regulatory Genes of JPJDXZF‐HCC Identified From Public Database

**FIGURE 1:**
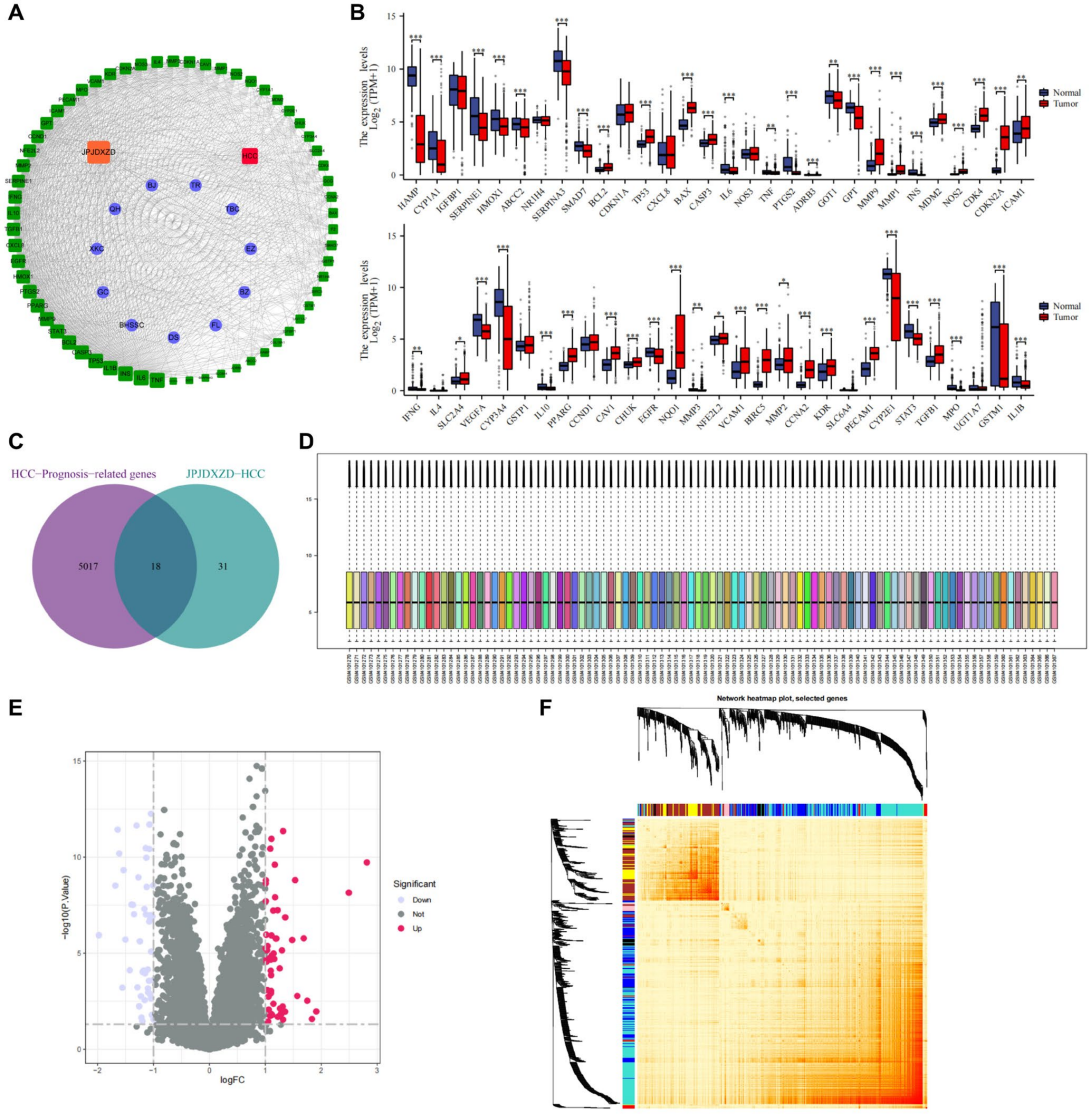
Identification of prognosis‐related and key genes of JPJDXZF in HCC based on public databases. (A) JPJDXZF‐active component‐HCC target PPI network. (B) Boxplot of HCC‐JPJDXZF related DEGs. (C) Intersection of survival prognosis genes from TCGA/GTEx and HCC‐JPJDXZF target genes. (D) Standardised sample distribution of GSE138178. (E) Volcano plot of DEGs in GSE138178. (F) Heatmap of expression changes of WGCNA clustered co‐expressed genes in each cluster. For boxplots, *n* denotes the number of samples per group. *p < 0.05, **p < 0.01, ***p < 0.001.

Through searches of the SymMap, TCMID, TCMSP, and TCM‐ID databases, and following ADME principal screening, a total of 1443 active components of JPJDXZF were identified, including 277 from Dangshen (DS), 124 from Fuling (FL), 144 from Baizhu (BZ), 96 from Ezhu (EZ), 5 from Tubiechong (TBC), 126 from Taoren (TR), 204 from Qinghao (QH), 114 from Xiakucao (XKC), 401 from Gancao (GC), 90 from Baihuasheshecao (BHSSC), and 16 from Biejia (BJ). In total, 435 corresponding drug targets were obtained, including 211 potential targets for DS, 27 for FL, 33 for BZ, 19 for EZ, 1 for TBC, 24 for TR, 107 for QH, 89 for XKC, 74 for GC, 62 for BHSSC, and 4 for BJ. Using Cytoscape 3.7.2, a JPJDXZF active component‐target network was constructed, comprising 653 nodes and 884 edges (Figure 1A). Disease Ontology enrichment analysis of the potential targets of JPJDXZF revealed that 61 genes were enriched in HCC. Clinical information and transcriptome expression data related to HCC were downloaded from the TCGA and GTEx databases and processed using R software. An expression matrix of the 61 JPJDXZF‐targeted HCC‐related genes was obtained. Based on a screening criterion of *p* < 0.05, 49 DEGs were identified, and their expression patterns were visualised using boxplots (Figure 1B). Prognostic analysis based on TCGA/GTEx data further showed that the intersection of 6703 prognosis‐related genes with the 49 DEGs resulted in 18 prognosis‐related DEGs (Figure 1C). To further validate key genes involved in HCC regulation, the GEO microarray dataset GSE138178 was used as an external validation cohort. This dataset included tumour tissues from 49 HCC patients and 49 paired adjacent normal tissues. After background correction and normalisation, the standardised distribution of samples was confirmed (Figure 1D). Differential expression analysis was performed using the criteria of *p* < 0.05 and |log₂FC| ≥ 1, identifying 533 upregulated and 300 downregulated DEGs, which were visualised in a volcano plot (Figure 1E). The expression matrix of DEGs from GSE138178 was subsequently subjected to WGCNA. A soft‐thresholding power of β = 4 was selected when the scale‐free topology fit index first exceeded 0.9. Clustering analysis of the 98 samples showed no obvious outliers, indicating suitability for network construction. Using *β* = 4, a gene co‐expression network was established, and genes were classified into seven distinct modules through dynamic tree cutting. Module‐trait relationship analysis demonstrated that the MEblue module was associated with HCC (|*r*| ≥ 0.70, *p* < 0.05), from which 1505 potential hub genes were identified. Finally, a heatmap was generated to illustrate the expression patterns of WGCNA‐clustered genes across different modules (Figure 1F).

### 3.2 Acquisition of the Core Target Genes of JPJDXZF in Treating HCC Based on TCGA and GEO Databases and Functional Analysis

**FIGURE 2:**
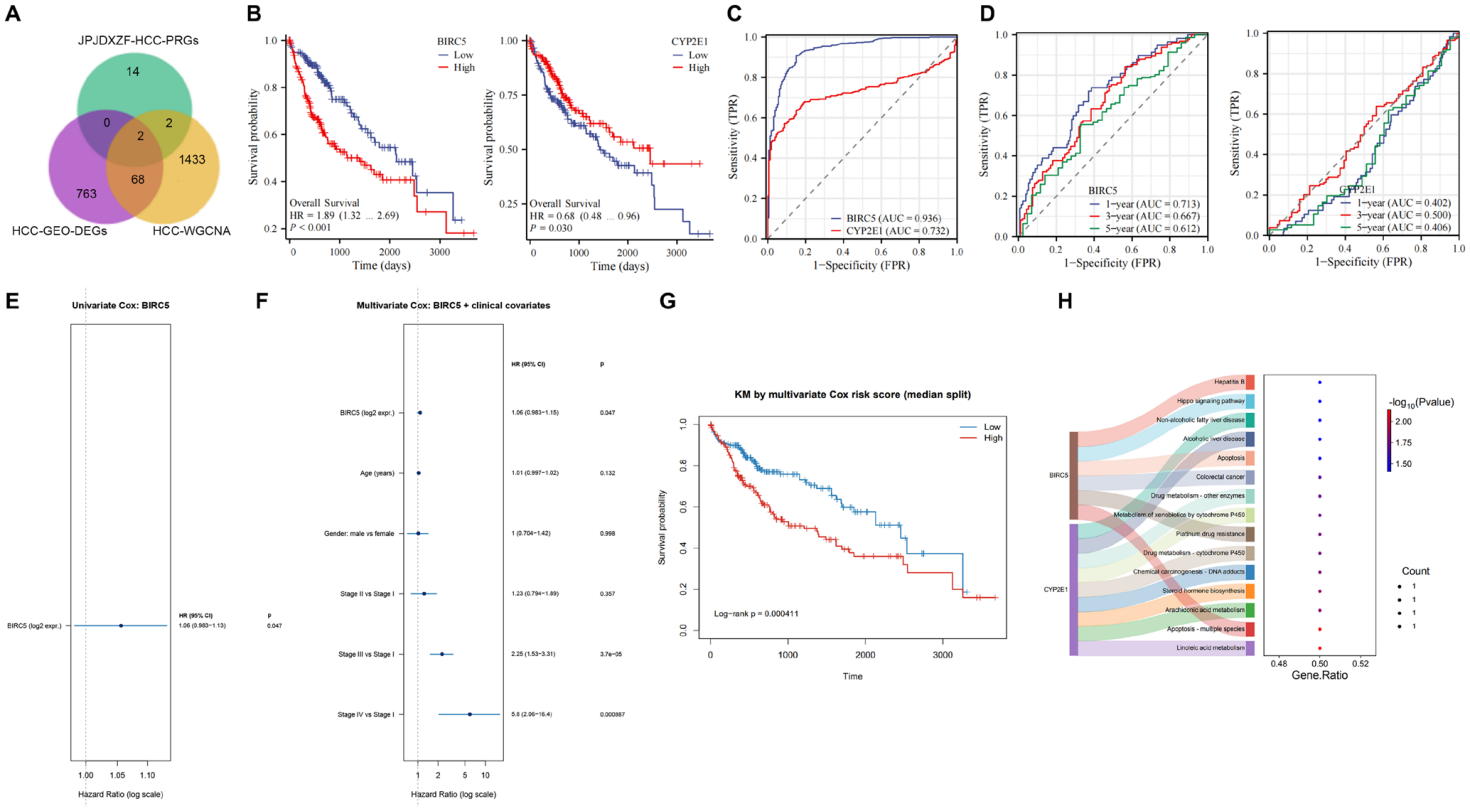
Acquisition of the core target genes of JPJDXZF in treating HCC based on TCGA and GEO databases and functional analysis. (A) Intersection Venn diagram. (B) Survival prognosis curves for core genes BIRC5 and CYP2E1. (C) ROC model for BIRC5 and CYP2E1. (D) Time‐dependent ROC curves showing the predictive accuracy of BIRC5 and CYP2E1 for 1‐, 3‐, and 5‐year survival. (E) Univariate Cox regression analysis of BIRC5 expression. (F) Multivariate Cox regression analysis incorporating BIRC5 expression and clinical covariates, including age, sex, and tumour stage. (G) Kaplan–Meier survival curves based on multivariate Cox risk score. (H) KEGG pathway enrichment analysis showing the pathways related to BIRC5 and CYP2E1. For survival and Cox analyses, *n* denotes the number of patients. Log‐rank and Cox proportional hazards models were applied. Error bars indicate SD. *p* < 0.05 was considered significant.

In the in‐depth analysis of the molecular mechanisms of HCC, we compared the 18 JPJDXZF‐related DEGs associated with HCC prognosis obtained earlier with 833 HCC‐GSE138178 related genes and 1505 HCC‐GSE138178‐WGCNA related genes. By importing them into the online Venn diagram tool InteractiVenn, we identified two potential targets for JPJDXZF in treating HCC: BIRC5 and CYP2E1 (Figure 2A). To better understand the prognostic implications of these two core genes in HCC patients, we observed that those with high BIRC5 expression had poorer survival outcomes compared to those with low expression. Conversely, patients with low CYP2E1 expression exhibited worse survival than those with high expression (Figure 2B). By constructing ROC curves for the core genes of HCC, we found that the AUC of BIRC5 reached 0.936, and the AUC of CYP2E1 reached 0.732, both indicating good diagnostic value (Figure 2C). Additionally, by constructing time‐dependent ROC curves, we evaluated the prognostic performance of BIRC5 and CYP2E1 at 1, 3, and 5 years (Figure 2D). For BIRC5, the AUC values were 0.713 (95% CI: 0.6416–0.7853) at 1 year, 0.667 (95% CI: 0.5908–0.7428) at 3 years, and 0.612 (95% CI: 0.5104–0.7127) at 5 years. CYP2E1 showed limited prognostic discrimination, with AUC values of 0.402 (95% CI: 0.3271–0.4775) at 1 year, 0.500 (95% CI: 0.4166–0.5830) at 3 years, and 0.406 (95% CI: 0.2999–0.5123) at 5 years. All time‐dependent ROC analyses were performed in a cohort of 424 HCC patients. These results indicate that BIRC5 exhibits moderate prognostic value, particularly for short‐term survival prediction, whereas CYP2E1 alone shows limited predictive performance. Then, Cox regression analyses were performed to evaluate the prognostic value of BIRC5. Univariate Cox regression analysis showed that higher BIRC5 expression was significantly associated with poorer overall survival in HCC patients (HR = 1.06, 95% CI: 0.98–1.13, *p* = 0.047, Figure 2E). After adjustment for clinical variables including age, sex, and tumour stage, multivariate Cox regression analysis showed that BIRC5 expression remained significantly associated with overall survival (Figure 2F). Furthermore, patients were stratified into high‐ and low‐risk groups based on the multivariate Cox risk score using the median value as the cutoff. Kaplan–Meier survival analysis revealed a significant difference in survival probability between the two groups, with the high‐risk group exhibiting markedly poorer outcomes (Figure 2G). Using Bioconductor package and clusterProfiler package in R, we performed KEGG pathway enrichment analysis on core targets of JPJDXZF in treating HCC and found that they were mainly concentrated in pathways such as Hippo pathway, linoleic acid metabolism, and apoptosis in multiple species. Hippo pathway is known to influence the regulation of various cancers, including HCC [20, 26, 27]. BIRC5, also known as Survivin, is an anti‐apoptotic protein and belongs to the inhibitor of apoptosis protein family [28]. Then KEGG pathway analysis showed the pathways related to BIRC5 and CYP2E1 in the treatment of HCC. Several pathways were enriched, including the Hippo pathway, apoptosis, drug metabolism, and cell cycle regulation (Figure 2H and Table S1). Notably, BIRC5 was identified as a downstream gene in the Hippo pathway, highlighting its role in HCC progression. These findings also suggest that the therapeutic effects of JPJDXZF may be mediated through a combination of these pathways, rather than by a single pathway alone. Therefore, we propose that JPJDXZF may exert therapeutic effects in HCC by regulating BIRC5 expression through the Hippo‐YAP/TAZ axis.

### 3.3 In Vitro Experiments to Explore the Effects of JPJDXZF on HCC Cell Proliferation and Migration

**FIGURE 3:**
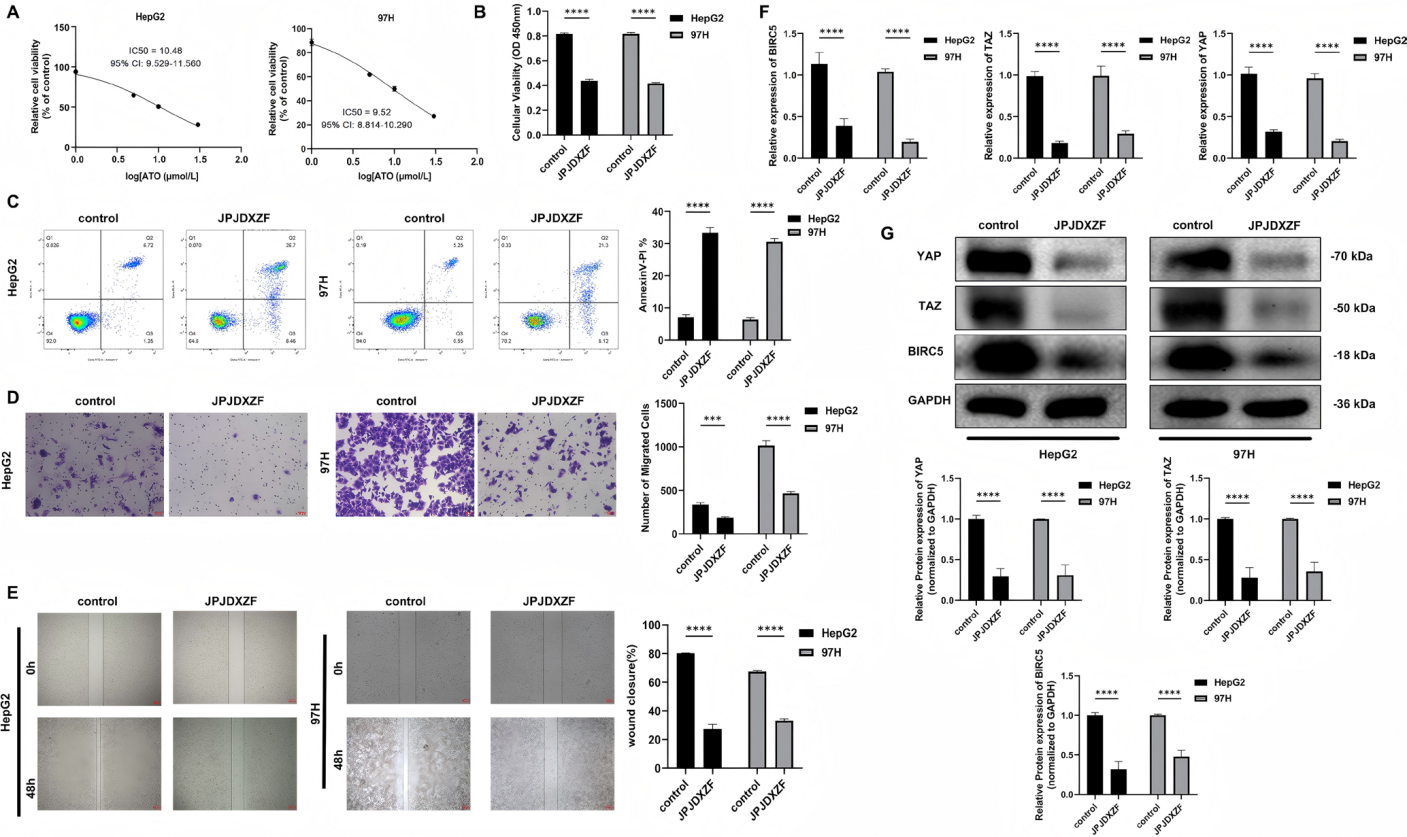
In vitro *experiments* to explore the effects of JPJDXZF on HCC cell proliferation and migration. (A) IC50 determination of JPJDXZF‐containing serum. IC50 values with 95% confidence intervals were calculated using a four‐parameter logistic model in GraphPad Prism. (B–E) Cell viability, apoptosis, and migration abilities of HepG2 and MHCC97‐H cells were evaluated using CCK‐8 assay, flow cytometry, Transwell assay, and wound healing (scratch) assay, respectively. (F) Relative mRNA expression levels of BIRC5, YAP, and TAZ were measured by qRT‐PCR. (G) Protein expression levels of BIRC5, YAP, and TAZ were assessed by Western blot analysis, normalised to GAPDH. Mean ± SD (*n* = 3); unpaired two‐tailed Student's *t*‐test. ***p < 0.001, ****p < 0.0001.

Next, we investigated the effects of JPJDXZF on the regulation of HCC cell proliferation and migration in vitro. First, we prepared JPJDXZF‐containing serum. HepG2 and MHCC97‐H cells were cultured and treated with JPJDXZF‐containing serum at concentrations of 0%, 1%, 5%, 10%, and 30%. After 24 h, cell viability was assessed using the CCK‐8 assay. The IC_50_ of JPJDXZF‐containing serum was 10.5% (95% CI: 9.53–11.56) for HepG2 cells and 9.5% (95% CI: 8.81–10.29) for MHCC97‐H cells (Figure 3A). Based on these results, a concentration of 10% was selected for subsequent experiments. Next, we treated HepG2 and MHCC97‐H cells with a 10% concentration of the JPJDXZF‐containing serum. We found that the viability of HepG2 and MHCC97‐H cells treated with JPJDXZF‐containing serum decreased, apoptosis increased, and migration ability declined (Figure 3B–E), suggesting that JPJDXZF may have anticancer activity. Furthermore, compared to the control group, mRNA and protein expression of BIRC5 and Hippo pathway effectors YAP and TAZ were downregulated in the JPJDXZF group (Figure 3F,G). In earlier bioinformatics analysis, it was found that high expression of BIRC5 in HCC was associated with poor patient survival prognosis, indicating that BIRC5 played a promoting role in HCC progression, possibly by inhibiting cell apoptosis to increase the survival capacity of tumour cells. These findings suggested that JPJDXZF treatment may be associated with suppression of YAP/TAZ activity and reduced BIRC5 expression, which is consistent with the activation of the Hippo pathway.

### 3.4 Organoid Experiments to Explore the Regulation of HCC Organoid Growth by JPJDXZF

**FIGURE 4:**
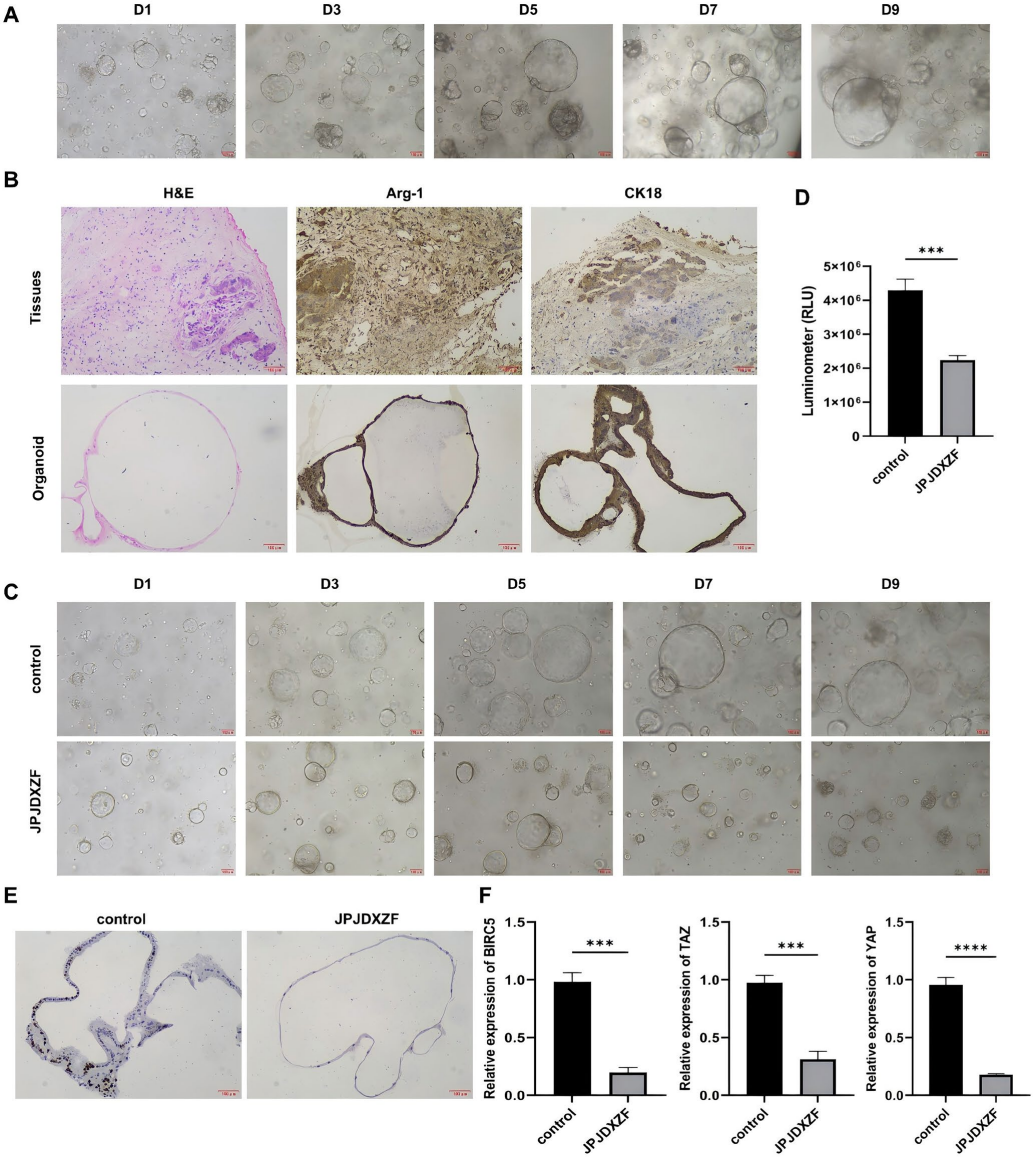
Organoid experiments to explore the regulation of HCC organoid growth by JPJDXZF. (A) Organoid growth images. (B) Pathological identification of organoids and tumour tissue (pathological changes detected by H&E staining, expression of Arg‐1 and CK18 detected by IHC). (C) Changes in organoid growth after treatment with JPJDXZF‐containing serum. (D) ATP activity detection after treatment of organoids with JPJDXZF‐containing serum. (E) Expression of tumour proliferation marker Ki67 detected by IHC after treatment of organoids with JPJDXZF‐containing serum. (F) Relative mRNA expression levels of BIRC5, YAP, and TAZ in organoids after treatment with JPJDXZF‐containing serum. Data are presented as mean ± SD (*n* = 3). Statistical significance was assessed using unpaired Student's *t*‐test. ***p < 0.001, ****p < 0.0001.

Next, we collected fresh HCC tissue samples and cultured HCC organoids. Microscopic observation showed the morphological features of HCC organoids at 1, 3, 5, 7, and 9 days. As the culture time extended, the diameter of the HCC organoids increased (Figure 4A). H&E staining further demonstrated the pathological similarity between HCC organoids and their source tissue (Figure 4B). In addition, IHC analysis showed high expression of Arg‐1 and CK18 in both HCC tissues and organoids, confirming that HCC organoids retained the pathological characteristics of the original tumours. These results indicated that we successfully constructed human HCC organoids. Furthermore, we explored the effects of JPJDXZF at the organoid level. HCC organoids were treated with JPJDXZF‐containing serum for 1, 3, 5, 7, and 9 days. Compared to the control group, the organoids' diameter in the JPJDXZF group decreased, ATP activity was reduced, and the tumour proliferation marker Ki67 was downregulated (Figure 4C–E), suggesting that JPJDXZF may have antitumor activity. Additionally, qRT‐PCR detection results showed that compared to the control group, BIRC5 and Hippo pathway effectors YAP and TAZ levels were downregulated in the JPJDXZF group (Figure 4F). These results indicated that JPJDXZF treatment was associated with suppressed growth of HCC organoids.

### 3.5 Animal Experiments Evaluating the Effects of JPJDXZF on Tumour Growth in Nude Mice

**FIGURE 5:**
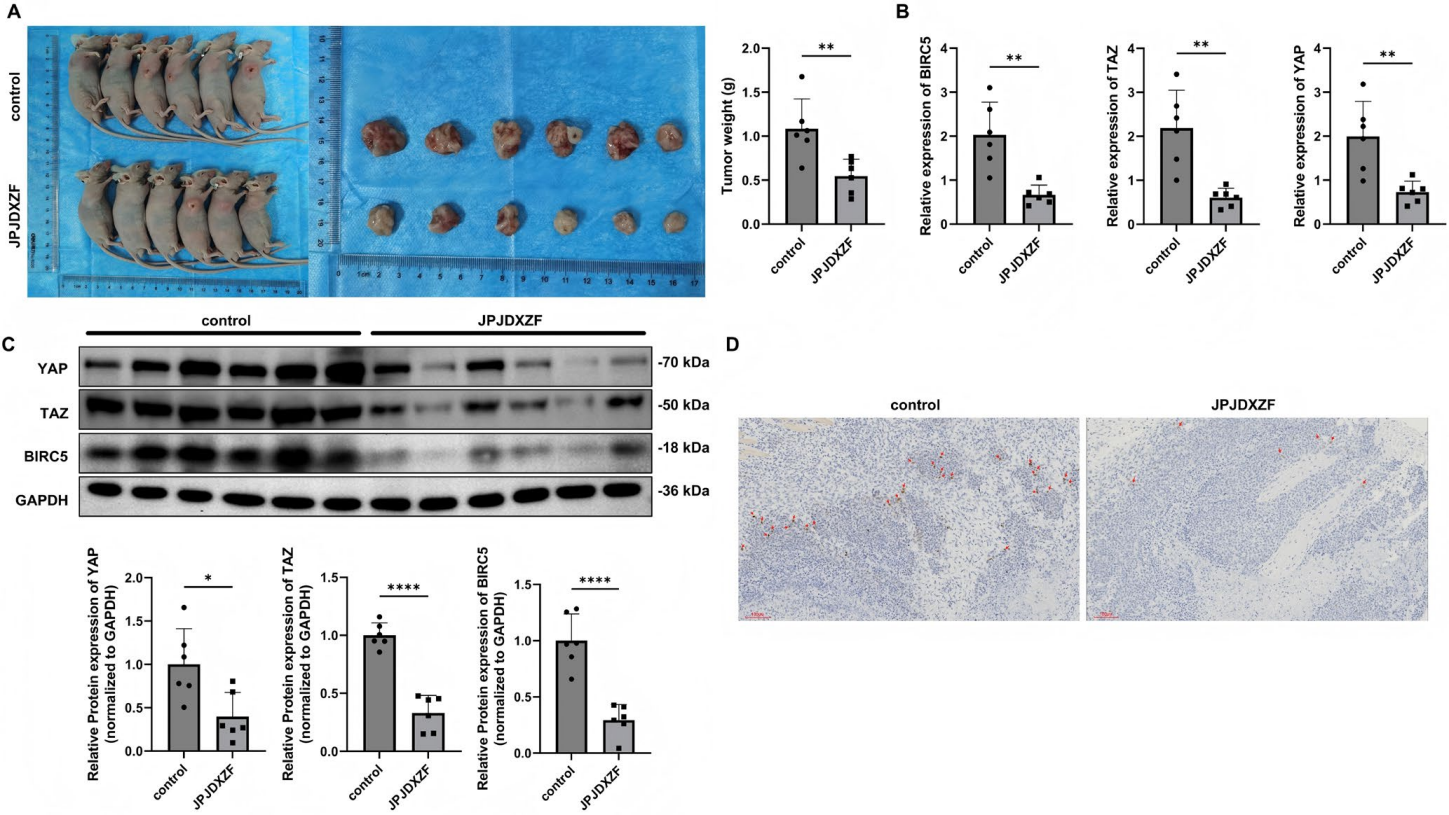
Animal experiments to evaluate the effects of JPJDXZF on tumour growth in nude mice. (A) Subcutaneous tumour‐bearing mice, tumour images, and tumour weight (*n* = 5). (B and C) Relative expression levels of BIRC5, YAP, and TAZ assessed by qRT‐PCR and Western blot, both normalised to GAPDH. (D) IHC analysis of the proliferation marker Ki67 in tumour tissues. Data are presented as mean ± SD (*n* = 3). Statistical significance was assessed using unpaired Student's *t*‐test. *p < 0.05, ****p < 0.0001 and **p < 0.01.

Additionally, we verified the function of JPJDXZF in nude mice. When subcutaneous tumours formed by the injection of HepG2 cells reached an average volume of 85 mm^3^, the mice were gavaged with JPJDXZF once a day for 15 consecutive days. We found that the tumours in the JPJDXZF group were smaller and lighter in weight than control group (Figure 5A). Furthermore, compared to control group, mRNA and protein expression of BIRC5 and Hippo pathway effectors YAP and TAZ were downregulated in the JPJDXZF group (Figure 5B,C). IHC results revealed that, compared to control group, the tumour proliferation marker Ki67 was downregulated in the JPJDXZF group (Figure 5D). Collectively, these results indicated that JPJDXZF was associated with inhibition of tumour growth in nude mice.

### 3.6 Effects of siMST1/2 Interference on JPJDXZF‐Treated HCC Cells

**FIGURE 6:**
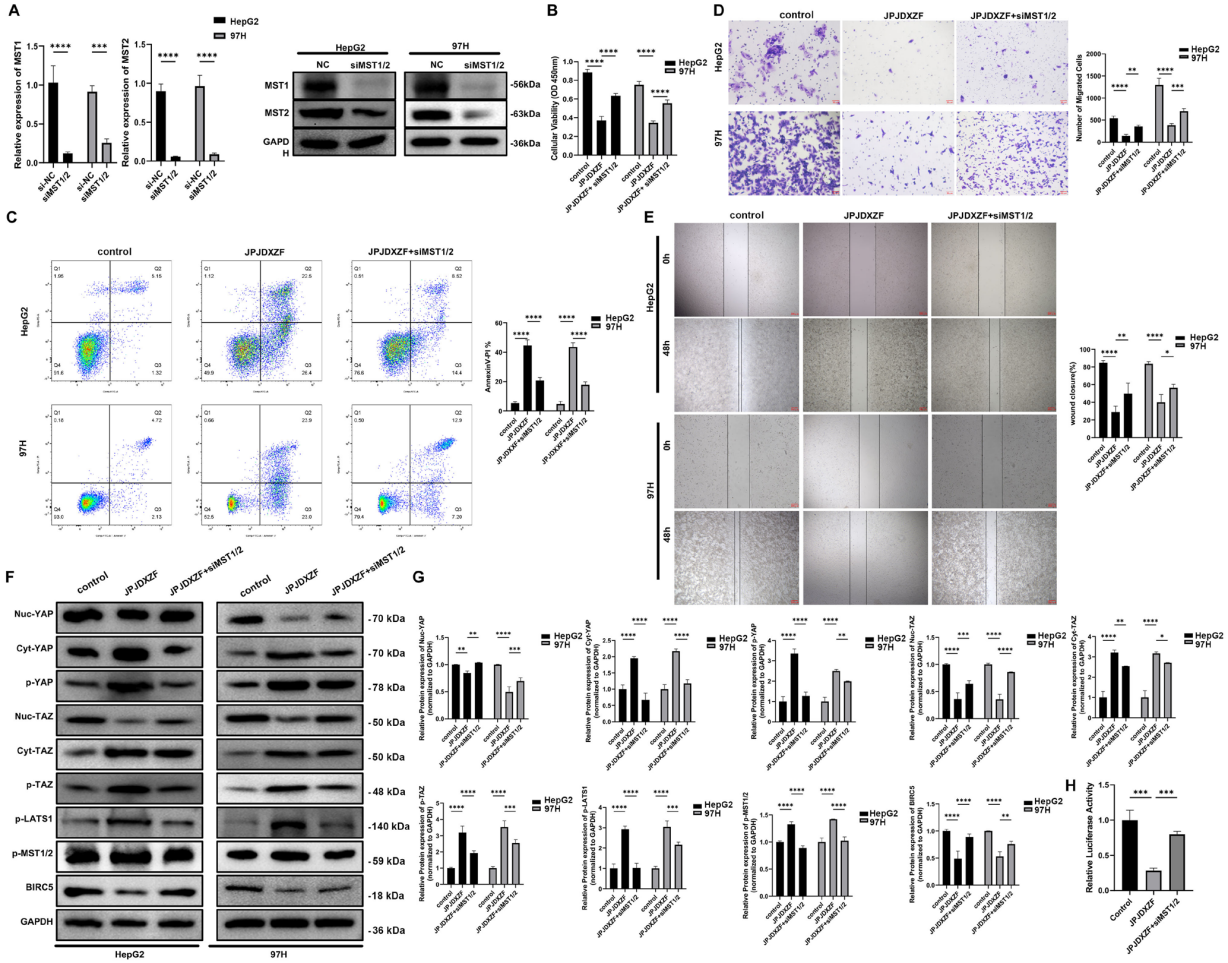
Effects of siMST1/2 interference on the regulatory role of JPJDXZF in HCC cells via the Hippo pathway. HepG2 and MHCC97‐H cells were transfected with si‐NC or siMST1/2 and treated with JPJDXZF‐containing serum as indicated. (A) qRT‐PCR and Western blot analyses verifying the knockdown efficiency of siMST1/2 in HepG2 and MHCC97‐H cells, both normalised to GAPDH. (B) Cell viability was assessed by CCK‐8 assay following different treatments. (C) Flow cytometric analysis of apoptosis. (D and E) Cell migratory ability was evaluated by Transwell and Wound healing assays. (F) Western blot analysis of nuclear and cytoplasmic YAP/TAZ, p‐TAZ, p‐LATS1, p‐MST1/2, and BIRC5, with GAPDH as the loading control. (G) Quantitative densitometric analysis of Western blot results, normalised to GAPDH. (H) TEAD luciferase reporter assay evaluating transcriptional activity downstream of the Hippo pathway. Data are presented as mean ± SD (*n* = 3). Statistical analysis was performed using one‐way ANOVA. *p < 0.05, **p < 0.01, ***p < 0.001, ****p < 0.0001.

To assess the efficiency of MST1/2 interference, HepG2 and MHCC97‐H cells were transfected with siMST1/2. qRT‐PCR and Western blot analyses showed that both mRNA and protein levels of MST1 and MST2 were markedly reduced in siMST1/2‐transfected cells compared with the si‐NC group (Figure 6A). Following JPJDXZF treatment, cell viability of HepG2 and MHCC97‐H cells was decreased, while interference with MST1/2 expression increased cell viability in JPJDXZF‐treated cells (Figure 6B). Flow cytometric analysis demonstrated that JPJDXZF treatment increased the proportion of apoptotic cells, whereas siMST1/2 transfection reduced apoptosis levels in both cell lines (Figure 6C). Transwell assays revealed that JPJDXZF treatment reduced the migratory capacity of HepG2 and MHCC97‐H cells, while this reduction was partially reversed in cells transfected with siMST1/2 (Figure 6D). Consistently, wound healing assay revealed slower wound closure in the JPJDXZF‐treated group, while enhanced wound closure was observed after MST1/2 interference (Figure 6E). Western blot analysis showed that JPJDXZF treatment decreased nuclear YAP and TAZ levels, increased cytoplasmic YAP/TAZ and phosphorylation of Hippo pathway components, and reduced BIRC5 expression, and these changes were attenuated by siMST1/2 interference (Figure 6F,G). In addition, TEAD luciferase reporter assays denoted the decreased transcriptional activity following JPJDXZF treatment, which was increased after MST1/2 interference (Figure 6H). Together, these data indicate that activation of the Hippo pathway contributes, at least in part, to the inhibitory effects of JPJDXZF on YAP/TAZ activity, BIRC5 expression, and HCC cell proliferation and migration.

### 3.7 In Vitro Experiments to Explore the Involvement of the Hippo Pathway in JPJDXZF‐Mediated Regulation of BIRC5 During HCC Development

**FIGURE 7:**
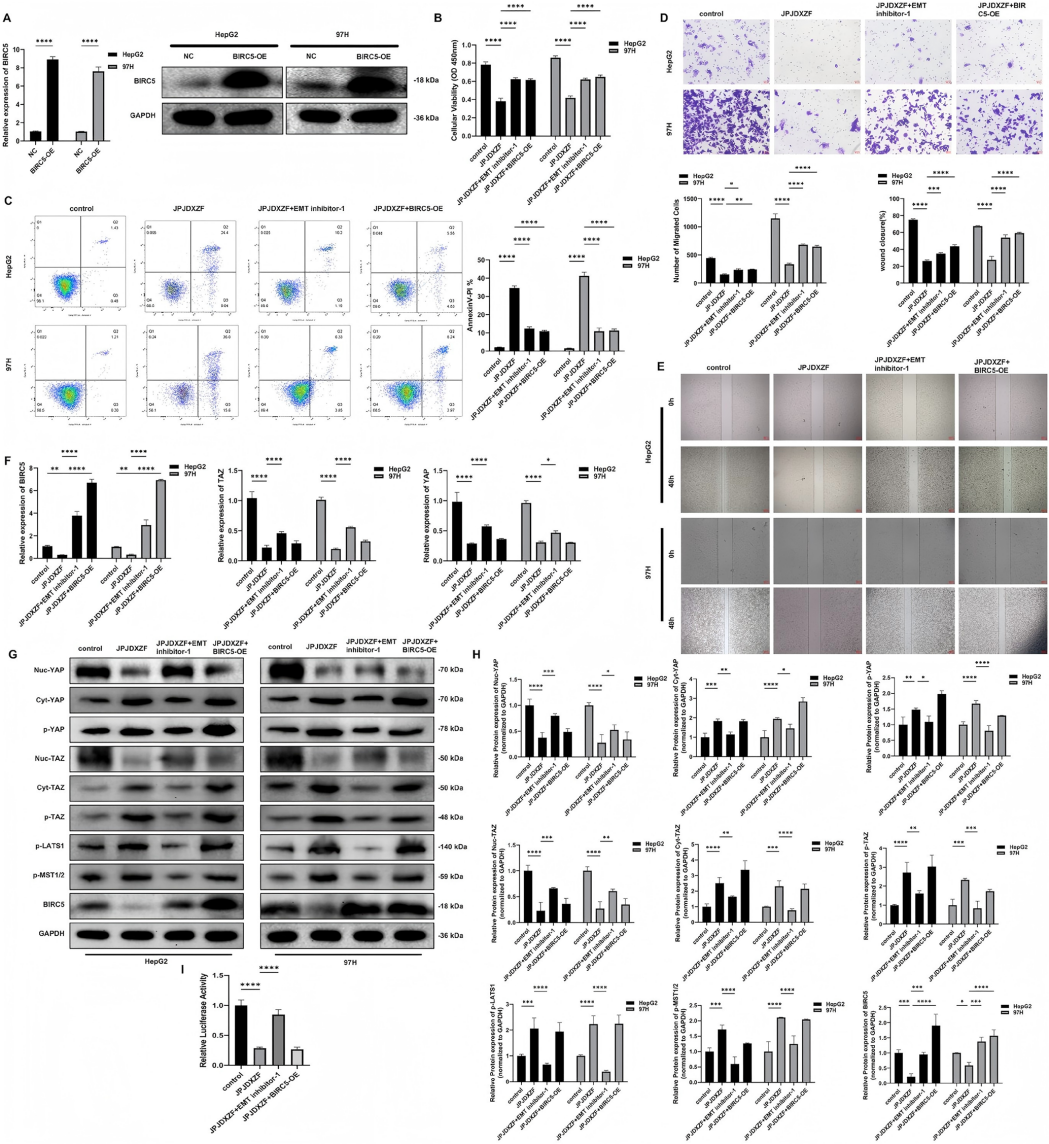
In vitro experiments investigating the regulation of BIRC5 expression by JPJDXZF via the Hippo pathway in HCC. (A) Verification of BIRC5 overexpression by qRT‐PCR and Western blot analysis, normalised to GAPDH. (B–E) Cell viability, apoptosis, and migratory capacity of HepG2 and MHCC97‐H cells were assessed using CCK‐8 assay, flow cytometry, Transwell assay, and wound healing assay, respectively. (F) Relative mRNA expression levels of BIRC5, YAP, and TAZ determined by qRT‐PCR. (G and H) Western blot analysis of total, nuclear, and cytoplasmic YAP/TAZ, phosphorylated YAP/TAZ, phosphorylated LATS1, phosphorylated MST1/2, and BIRC5 (GAPDH was used as internal reference). (I) The transcriptional activity downstream of the Hippo pathway was evaluated by the luciferase reporter assay. Data are presented as mean ± SD (*n* = 3). Statistical analysis was performed using one‐way ANOVA. *p < 0.05, **p < 0.01, ***p < 0.001, ****p < 0.0001.

Finally, we investigated at the cellular level whether JPJDXZF regulates BIRC5 expression through the Hippo pathway during HCC progression. BIRC5 was first overexpressed in HepG2 and MHCC97‐H cells by transfection with a BIRC5‐OE plasmid. Compared with the NC group, both mRNA and protein levels of BIRC5 were markedly increased in the BIRC5‐OE group (Figure 7A). After transfection with NC or BIRC5‐OE, HepG2 and MHCC97‐H cells were further treated with JPJDXZF‐containing serum or the Hippo pathway inhibitor EMT inhibitor‐1. Compared with the control group, JPJDXZF treatment reduced cell viability, increased apoptosis, and suppressed cell migration in both cell lines. EMT inhibitor‐1 treatment or BIRC5 overexpression partially restored cell viability and migratory capacity and reduced apoptosis (Figure 7B–E). At the transcriptional level, JPJDXZF treatment reduced the mRNA expression of BIRC5, YAP, and TAZ relative to the control group, whereas EMT inhibitor‐1 treatment or BIRC5 overexpression resulted in increased mRNA levels of these genes (Figure 7F). At the protein level, JPJDXZF treatment decreased BIRC5 expression, reduced nuclear YAP and TAZ levels, and increased cytoplasmic YAP and TAZ levels, accompanied by elevated phosphorylation of YAP, TAZ, LATS1, and MST1/2 compared with the control group. These changes were partially reversed by EMT inhibitor‐1 treatment or BIRC5 overexpression, as confirmed by densitometric analysis (Figure 7G,H). In addition, TEAD luciferase reporter assays showed reduced transcriptional activity following JPJDXZF treatment, while this effect was partially reversed by EMT inhibitor‐1 or BIRC5 overexpression (Figure 7I). Collectively, these results indicate that JPJDXZF treatment is associated with changes in Hippo pathway‐related signalling and BIRC5 expression, accompanied by altered proliferation, apoptosis, and migration of HCC cells.

## 4 Discussion

HCC remains a major global health challenge, characterised by limited therapeutic options and an unfavourable prognosis [29]. Although JPJDXZF has shown therapeutic potential in HCC, its underlying molecular mechanisms have not been fully elucidated. In the present study, we combined NP and bioinformatics analyses with cellular, patient‐derived organoid, and animal experiments to investigate the potential mechanisms of JPJDXZF in HCC, with particular attention to the key HCC‐associated gene BIRC5 and Hippo pathway–related signalling. Our findings suggest that JPJDXZF may exert antitumor effects in HCC, at least in part, through modulation of BIRC5 expression in association with Hippo pathway activity. This study provides experimental evidence supporting an association between JPJDXZF treatment and Hippo pathway–related regulation in HCC.

NP serves as a crucial tool, offering a holistic perspective on the interactions among compounds, genes, and diseases, thereby facilitating advancements in disease treatment and management [30]. TCM has developed a new approach with holistic and systemic ‘network targets’, which is the core theory and method of NP [31]. In this study, NP analysis identified 1443 active components of JPJDXZF, with 435 corresponding targets. DO analysis revealed that 61 genes were enriched in HCC. We selected 49 HCC‐JPJDXZF‐related genes from the dataset. Venn diagram showed that there were 18 intersecting genes between HCC prognosis‐related genes and HCC‐JPJDXZF‐related genes. Bioinformatics is one of the latest fields in biological research and is widely regarded as using mathematical, statistical, and computational methods to process and analyse biological data [32]. Tumour bioinformatics is crucial in cancer research and precision medicine [33]. In this study, we further analysed HCC at the gene expression level and identified 533 upregulated and 300 downregulated DEGs in the external test set GSE138178. The MEblue co‐expression module showed a significant association with HCC, with a correlation coefficient |*r*| ≥ 0.70 and *p* < 0.05, indicating that there may be HCC hub genes in the MEblue module, and ultimately, 1505 potential hub genes were identified. These bioinformatics analyses provided a rational framework for prioritising key candidate genes for subsequent experimental validation, rather than representing isolated statistical associations.

In further analysing the molecular mechanisms of HCC, the Venn diagram successfully identified two potential targets for JPJDXZF in treating HCC: BIRC5 and CYP2E1. BIRC5 is a conserved eukaryotic protein essential for cell division and capable of preventing cell death [34]. It is overexpressed in most cancers, promoting their clonal expansion, and has been considered a potential anticancer target for nearly two decades [35]. Elevated levels of BIRC5 are associated with poor clinical prognosis, tumour recurrence, and treatment resistance [36]. In HCC, high expression of BIRC5 and the infiltration level of myeloid‐derived suppressor cells were reportedly linked to a poorer prognosis in HCC patients [37]. Furthermore, BIRC5 inhibited HCC by blocking the PPARγ pathway and regulating cuprotosis, which may have therapeutic implications for HCC [38]. CYP2E1 is one of the 57 cytochrome P450 genes in the human genome and is highly conserved [39]. Additionally, CYP2E1 plays a key role in liver carcinogenesis, drug toxicity, and liver diseases [40]. It is reported to be actively involved in the pathogenesis of HCC and could serve as an emerging agent in HCC development and treatment [41]. Furthermore, CYP2E1 deficiency mediated bile acid‐induced malignant growth of HCC cells [42]. Collectively, these findings support the biological relevance of BIRC5 and CYP2E1 as potential targets through which JPJDXZF may influence HCC progression.

KEGG pathway enrichment analysis of core targets of JPJDXZF in treating HCC revealed that they were mainly concentrated in pathways such as Hippo pathway, linoleic acid metabolism, and apoptosis in multiple species. Hippo pathway is an evolutionarily conserved signalling cascade [43]. By integrating various mechanical, biochemical, and stress signals, Hippo pathway coordinates cell proliferation, survival, differentiation, and mechanics, thereby regulating organ development, homeostasis, and regeneration [44]. Research has shown that dysregulation of the Hippo signalling was closely related to tumour occurrence and progression [45]. Therefore, targeting Hippo pathway has become a promising cancer treatment strategy. In HCC, the regulatory role of Hippo pathway involved multiple aspects, including cell proliferation, invasion and metastasis, tumour resistance, metabolic reprogramming, immune regulation, and autophagy [46]. In this study, through analysis, we found that the core targets of JPJDXZF in treating HCC were mainly concentrated in Hippo pathway, and BIRC5 emerged as a downstream effector within the Hippo pathway–associated regulatory network [47]. The ROC analysis showed a high AUC value for BIRC5, indicating strong discriminatory performance between tumour and non‐tumour samples. Accordingly, our results suggest that JPJDXZF may participate in HCC treatment by modulating BIRC5 expression in association with the Hippo–YAP/TAZ axis, rather than through a single linear pathway.

Importantly, a previous experimental study has demonstrated that activation of YAP/TAZ signalling can enhance the expression of anti‐apoptotic genes, including BIRC5, thereby promoting cell survival and proliferation in cancer models [23]. Consistent with these reports, our experimental data show that JPJDXZF treatment suppresses YAP/TAZ nuclear localisation and TEAD‐dependent transcriptional activity, accompanied by reduced BIRC5 expression across cellular, organoid, and in vivo models. Furthermore, genetic manipulation of core Hippo kinases partially reversed the effects of JPJDXZF on YAP/TAZ activity and BIRC5 expression, providing functional support for the involvement of Hippo signalling in this regulatory axis. However, unlike studies focusing on direct transcriptional regulation, the present work primarily provides pathway‐level and functional evidence, and whether BIRC5 represents a direct YAP/TAZ transcriptional target in HCC requires further investigation.

Hippo signalling is not the only pathway involved in the biological effects of JPJDXZF. KEGG enrichment analysis identified several additional pathways related to apoptosis and metabolism, indicating that JPJDXZF may act through a broader, multi‐pathway regulatory network. Moreover, EMT inhibitor‐1 was used as a pharmacological probe to further examine Hippo pathway involvement; however, previous studies have shown that this compound can influence multiple signalling pathways. Accordingly, the evidence supporting Hippo pathway activation in the present study relies primarily on genetic manipulation of core Hippo kinases rather than pharmacological intervention alone.

TCM has been used in various stages of HCC treatment, even before tumour formation [48]. It exhibits multi‐target and synergistic intervention effects on HCC [49]. Wu et al. [13] reported that the Yiqi Jianpi Jiedu formula primarily exerts its anti‐HCC effects through multiple bioactive components as well as various pathways and targets. Zhang et al. [15] found that the therapeutic target genes of Jianpi Jiedu decoction are mainly involved in the metabolism and apoptosis of HCC, and the prognosis of HCC was closely related to genes such as CCNB1, NQO1, NUF2, and CHEK1. Additionally, Jianpi Jiedu decoction could promote the effects of fasting and diarrhoea pre‐treatment in HCC rats via regulating ABCC2 and OATP1B2 expressions in liver and cancer tissues [50]. Building on these studies, our findings further suggest that JPJDXZF suppresses HCC progression through coordinated regulation of tumour proliferation and survival signalling. Moreover, organoids more closely simulate the in vivo environment and cellular interactions, replicating the spatial organisation of cell surface receptors and gene expression, and may become an important tool for selecting treatment methods and assessing tumour responses to therapy [51]. In this study, cellular, organoid, and animal experiments consistently demonstrated that JPJDXZF‐containing serum reduced HCC cell viability and migration, decreased organoid diameter and ATP activity, downregulated Ki67 expression, and inhibited tumour growth in nude mice, accompanied by reduced expression of BIRC5 and Hippo pathway effectors YAP and TAZ. These results strengthen the translational relevance of our findings by linking molecular regulation with phenotypic outcomes across multiple experimental models. Notably, the consistent regulation of Hippo pathway activity and BIRC5 expression across cellular, organoid, and in vivo models further supports the robustness of this proposed regulatory mechanism.

The anticancer effects of JPJDXZF in HCC may involve Hippo signalling and its downstream effector BIRC5. Consequently, we further verified the roles of Hippo pathway and BIRC5 by adding Hippo pathway inhibitor EMT inhibitor‐1 and overexpressing BIRC5. Mechanistically, we found that JPJDXZF may activate Hippo pathway to affect the expression of effectors YAP and TAZ, thereby regulating BIRC5 expression to participate in the proliferation and apoptosis of HCC cells. This study is the first to experimentally associate JPJDXZF treatment with activation of Hippo pathway–related signalling in HCC.

Despite these findings, the present study has certain limitations. In particular, direct manipulation of the Hippo pathway was not performed in the in vivo model. Further studies incorporating targeted genetic or pharmacological modulation will be required to more comprehensively define pathway dependency under physiological conditions. In addition, the preparation of drug‐containing serum was based on a relatively small number of animals, and studies involving larger cohorts will be beneficial for further strengthening the reliability of the conclusions. Furthermore, EMT inhibitor‐1 was used in this study as a pharmacological probe to support Hippo pathway involvement. Although this compound was originally identified as a small‐molecule inhibitor (C19) capable of modulating the MST/LATS–YAP/TAZ axis and promoting TAZ degradation, it has also been reported to affect other signalling pathways. Therefore, the pharmacological data should be interpreted with caution, and the mechanistic conclusions regarding Hippo pathway activation are primarily supported by genetic manipulation of core Hippo kinases rather than by pharmacological intervention alone.

## 5 Conclusion

In this study, we explored the molecular mechanisms underlying the anti‐HCC effects of JPJDXZF at the cellular, organoid, and animal levels based on NP and bioinformatics analyses. Our findings suggest that JPJDXZF exerts antitumor effects partly through modulation of Hippo pathway–related signalling and regulation of the key HCC‐associated gene BIRC5. This study provides a mechanistic framework for the therapeutic application of JPJDXZF and offers insights for further investigation of Hippo signalling in HCC.

## Author Contributions

**Bin Li** and **Chong Zhong:** conceptualization, validation, and resources. **Han‐Qian Shi**, **Rui Luo**, **Zi‐Qi Zhang**, **Xiao‐Chen Dong**, **Xiao‐Hua Li**, and **Shi‐Qin Ye:** methodology and investigation. **Bin Li**, **Han‐Qian Shi**, **Rui Luo**, **Zi‐Qi Zhang**, **Xiao‐Chen Dong**, **Xiao‐Hua Li**, **Shi‐Qin Ye**, **Chong Zhong:** data curation, formal analysis, writing – original draft, and writing – review and editing.

## Funding

This study was supported by the ‘Yulong Talent’ Cultivation Program of Beijing University of Chinese Medicine Shenzhen Hospital (Longgang) (Grant number 2023‐BUCMSZYLRC18).

## Ethics Statement

All procedures performed in studies involving human participants were in accordance with the ethical standards of the institutional and/or national research committee and with the 1964 Helsinki Declaration and its later amendments or comparable ethical standards. Written informed consent was obtained from all patients who provided samples, and the study was approved by The First Affiliated Hospital of Guangzhou University of Chinese Medicine (approval number KY‐2024‐369, approval date 20241105). All animal experiments have been approved by Guangzhou Forevergen Medical Laboratory Animal Center (approval number IACUC‐AEWC‐F240601016, approval date 20240601). Animal experiments were performed in accordance with ARRIVE guidelines and the IACUC Handbook (third edition).

## Consent

The authors have nothing to report.

## Conflicts of Interest

The authors declare no conflicts of interest.

## Supporting information


**Table S1:** KEGG pathway enrichment analysis of core targets of JPJDXZF in HCC.

## Data Availability

The data generated in the present study are included in the figures and/or tables of this article.
