# Supplementary material for: Jianpi Jiedu Xiaozheng Fang Regulates Hepatocellular Carcinoma Proliferation and Metastasis Based on Network Pharmacology
Source: J Cell Mol Med. 2026 Mar 7;30(5):e71040. doi: 10.1111/jcmm.71040 (PMC12967627; doi:10.1111/jcmm.71040)
Supplement: Supplementary file 1 — Table S1: KEGG pathway enrichment analysis of core targets of JPJDXZF in HCC. [file JCMM-30-e71040-s001.docx]

**Supplementary Table 1**. KEGG pathway enrichment analysis of core targets of JPJDXZF in HCC

| **Description** | **GeneRatio** | **BgRatio** | **pvalue** | **p.adjust** | **qvalue** | **geneID** | **Count** |
| --- | --- | --- | --- | --- | --- | --- | --- |
| Linoleic acid metabolism | 1/2 | 30/8223 | 0.007283739 | 0.035374993 | 0.004380804 | CYP2E1 | 1 |
| Apoptosis - multiple species | 1/2 | 32/8223 | 0.007768375 | 0.035374993 | 0.004380804 | BIRC5 | 1 |
| Arachidonic acid metabolism | 1/2 | 61/8223 | 0.0147823 | 0.035374993 | 0.004380804 | CYP2E1 | 1 |
| Steroid hormone biosynthesis | 1/2 | 62/8223 | 0.015023716 | 0.035374993 | 0.004380804 | CYP2E1 | 1 |
| Chemical carcinogenesis - DNA adducts | 1/2 | 69/8223 | 0.016712798 | 0.035374993 | 0.004380804 | CYP2E1 | 1 |
| Drug metabolism - cytochrome P450 | 1/2 | 72/8223 | 0.017436246 | 0.035374993 | 0.004380804 | CYP2E1 | 1 |
| Platinum drug resistance | 1/2 | 73/8223 | 0.017677337 | 0.035374993 | 0.004380804 | BIRC5 | 1 |
| Metabolism of xenobiotics by cytochrome P450 | 1/2 | 78/8223 | 0.018882345 | 0.035374993 | 0.004380804 | CYP2E1 | 1 |
| Drug metabolism - other enzymes | 1/2 | 80/8223 | 0.019364141 | 0.035374993 | 0.004380804 | CYP2E1 | 1 |
| Colorectal cancer | 1/2 | 86/8223 | 0.020808819 | 0.035374993 | 0.004380804 | BIRC5 | 1 |
| Apoptosis | 1/2 | 136/8223 | 0.032806393 | 0.044218025 | 0.005475916 | BIRC5 | 1 |
| Alcoholic liver disease | 1/2 | 142/8223 | 0.034241132 | 0.044218025 | 0.005475916 | CYP2E1 | 1 |
| Non-alcoholic fatty liver disease | 1/2 | 155/8223 | 0.03734608 | 0.044218025 | 0.005475916 | CYP2E1 | 1 |
| Hippo signaling pathway | 1/2 | 157/8223 | 0.03782332 | 0.044218025 | 0.005475916 | BIRC5 | 1 |
| Hepatitis B | 1/2 | 162/8223 | 0.039015904 | 0.044218025 | 0.005475916 | BIRC5 | 1 |
